# Descending serotonergic controls regulate inflammation-induced mechanical sensitivity and methyl-CpG-binding protein 2 phosphorylation in the rat superficial dorsal horn

**DOI:** 10.1186/1744-8069-4-35

**Published:** 2008-09-15

**Authors:** Sandrine M Géranton, Vincenza Fratto, Keri K Tochiki, Stephen P Hunt

**Affiliations:** 1 Department of Cell and Developmental Biology, University College London, Medawar Building, London, WC1E 6BT, UK; 2 Department of Pharmacobiology, University of Calabria, 87036 Arcavacata di Rende (Cs), Italy

## Abstract

**Background:**

Regulation of pain states is, in part, dependent upon plastic changes in neurones within the superficial dorsal horn. There is also compelling evidence that pain states are under the control of descending projections from the brainstem. While a number of transcription factors including Methyl-CpG-binding protein 2 (MeCP2), Zif268 and Fos have been implicated in the regulation of dorsal horn neurone sensitization following injury, modulation of their activity by descending controls has not been investigated.

**Results:**

Here, we describe how descending controls regulate MeCP2 phosphorylation (P-MeCP2), known to relieve transcriptional repression by MeCP2, and Zif268 and Fos expression in the rat superficial dorsal horn, after CFA injection into the hind paw. First, we report that CFA significantly increased P-MeCP2 in Lamina I and II, from 30 min post injection, with a maximum reached after 1 h. The increase in P-MeCP2 paralleled that of Zif268 and Fos, and P-MeCP2 was expressed in large sub-populations of Zif268 and Fos expressing neurones. Serotonergic depletion of the lumbar spinal cord with 5,7 di-hydroxytryptamine creatinine sulphate (5,7-DHT) reduced the inflammation evoked P-MeCP2 in the superficial dorsal horn by 57%, and that of Zif268 and Fos by 37.5% and 30% respectively. Although 5,7-DHT did not change primary thermal hyperalgesia, it significantly attenuated mechanical sensitivity seen in the first 24 h after CFA.

**Conclusion:**

We conclude that descending serotonergic pathways play a crucial role in regulating gene expression in the dorsal horn and mechanical sensitivity associated with an inflammatory pain state.

## Background

The development and maintenance of pain states are dependant upon plastic changes in neurones of the superficial dorsal horn that are thought to be under the control of descending pathways originating in the brainstem [1,2]. The transcription factors Fos, Zif268 and Methyl-CpG-binding protein 2 (MeCP2) have been implicated in dorsal horn plasticity yet their dependence on descending controls for their full activation has not been explored.

MeCP2 is a transcriptional repressor that regulates activity-dependent gene transcription and is critical for normal neurological function. Mutations in human MeCP2 result in the neurodevelopmental disorder Rett syndrome [3,4]. However, we know very little about the physiological role of MeCP2 in the central nervous system. MeCP2 regulates gene transcription by binding to methylated CpG dinucleotides and recruiting co-repressors such as histone deacetylases to promote chromatin compaction and reduce access of transcription factors to promoter DNA [5]. *In vitro*, phosphorylation of MeCP2 at S421 was shown to lead to the displacement of MeCP2 from the BDNF promoter resulting in an increase in BDNF expression [6]. We recently showed that MeCP2 was phosphorylated in the rat superficial dorsal horn after induction of peripheral joint inflammation, leading to an increase in expression of a family of genes under transcriptional control by MeCP2. These included the serum- and glucocorticoid- regulated kinase (SGK1) which we found was involved in the induction of pain states [7]. Numerous studies also support a role for Fos and Zif268, a transcription factor essential for the maintenance of longer term LTP in the hippocampus, in the initiation and maintenance of pain states [8,9].

Descending controls have been shown to play a crucial role in the regulation of pain states [10]. We therefore considered the possibility that the changes in gene expression that follow activation of transcription factors in the dorsal horn are the result of converging patterns of activity and not simply a response to primary afferent stimulation. Serotonergic controls are essential for the maintenance of neuropathic pain states and the full development of ERK activation, a kinase essential for the development of central sensitization [11,12]. We have therefore investigated the contribution of serotonergic inputs to the activation of transcription factors MeCP2, Zif268 and Fos as well as on the development of mechanical and thermal hyperalgesia induced by peripheral inflammation. A number of serotonergic receptors and descending serotonergic pathways have been implicated in the maintenance of pain states [13]. We therefore investigated the regulation of transcription factors after depletion of spinal 5-HT using 5,7 di-hydroxytryptamine creatinine sulphate (5,7-DHT). We found that serotonergic controls participated in both the activation of transcription factors and the mechanical hypersensitivity that develops in the first hours after inflammation.

## Methods

### Animals' preparation

All procedures complied with the UK Animals (Scientific Procedures) Act 1986. Experiments were carried out on male Sprague-Dawley rats (180–250 g body weight) from the colony at University College London. All efforts were made to minimise animal suffering and to reduce the number of animals used. Inflammation was induced by injection of Complete Freund's Adjuvant (CFA, Sigma, Pool, UK; 50 μl) in the left hind paw, under halothane anaesthesia.

### Immunohistochemistry

Rats were perfused as described [7] and lumbar spinal cord was dissected out and cut on a freezing microtome set at 40 μm. The antibodies were revealed by diaminobenzidinetetrahydrochloride (DAB, peroxidase substrate) or fluorescent immunohistochemistry, as appropriate. For DAB reactions, sections were left to incubate with primary antibodies for 48 h at 4°C (P-MeCP2, S421; 1:10,000; a gift from Zhaolan Zhou and Michael Greenberg) or overnight at room temperature (Fos, Oncogene, 1:60,000 and Zif268, Santa Cruz sc-189, 1:10,000) followed by conventional DAB protocol. For MeCP2 fluorescent immunohistochemistry, we followed a protocol of TSA amplification as described previously [7]. For double labelling with Zif268 and Fos, sections stained for P-MeCP2 were left for 24 h at room temperature in a solution of Zif268 (1:5000) or Fos (1:5000) antibody. Direct secondary was used at a concentration of 1:500 (Anti-rabbit Alexa 594). Both NeuN (a specific neuronal marker; Chemicon; 1:1000, 24 h room temperature) and GFAP (Dako, 1:4000, 24 h room temperature) were revealed using direct secondary as described above. 5-HT (Chemicon; 1:75; 24 h room temperature) was revealed using a biotinylated secondary antibody (1:500 for 2 h) followed by streptavidin conjugated Cy3 (Jackson Laboratories; 1:4 000; 45 min). Controls for immunohistochemistry included omitting the first or second primary antibodies.

### Intrathecal administration of 5,7 di-hydroxytryptamine

5,7 di-hydroxytryptamine creatinine sulphate (5,7-DHT; Sigma-Aldrich) was administered *via *intrathecal injection as described [11]. To prevent toxicity to noradrenergic neurones, rats received desipramine (a noradrenaline reuptake inhibitor; Sigma-Aldrich; 25 mg/kg) 1 h prior to 5,7-DHT administration. Animals received either 10 μl of 5,7-DHT (60 μg) or saline 0.9%.

### Behavioural assay

For each behavioural assay, animals were habituated over a period of 3 consecutive days by recording a series of baseline measurements. Animals were allowed to habituate to the experimental room for 10–15 min before testing began. Four readings were collected per animal with an inter-stimulus interval of 5 min. Thermal withdrawal thresholds were determined as described by Hargreaves [14] using the Plantar Test Apparatus, Stoelting, IL, US. Responses to mechanical stimuli were measured with the Automatic Von Frey apparatus from Ugo-Basile (Italy). The ramp was set to reach the maximum stimulus (50 g) in 20 sec.

### Data analysis

For all data, statistical analysis was carried out on the raw data using SPSS PC+. Changes in P-MeCP2, Zif268 and Fos expression were quantified by immunohistochemistry. Immunopositive cells were counted in the dorsal horn of CFA-injected animals (ipsi, 5 sections with maximum response per animal). Data were analysed by general linear model univariate or multivariate test followed by LSD post-hoc tests or Student's t-test, as appropriate.

For behaviour, the significance of any differences in mechanical and thermal thresholds was assessed using repeated measures two-way ANOVA (SPSS PC+). A significant effect on the main factor(s) was taken as the criterion for progressing to post-hoc testing. 'Time' was treated as 'within subjects' factor and 'treatment' was treated as a 'between' subjects factor. Comparisons of changes in different groups of rats were carried out on time-matched samples. When appropriate, the Greenhouse – Geisser 'ε' correction was applied to correct for any violation of sphericity of the variance – covariance matrix. Statistical significance was set at *P *< 0.05.

## Results

### MeCP2 phosphorylation after CFA injection in the hind paw is maximum in lamina I-II and 1 h after CFA injection

**Figure 1:**
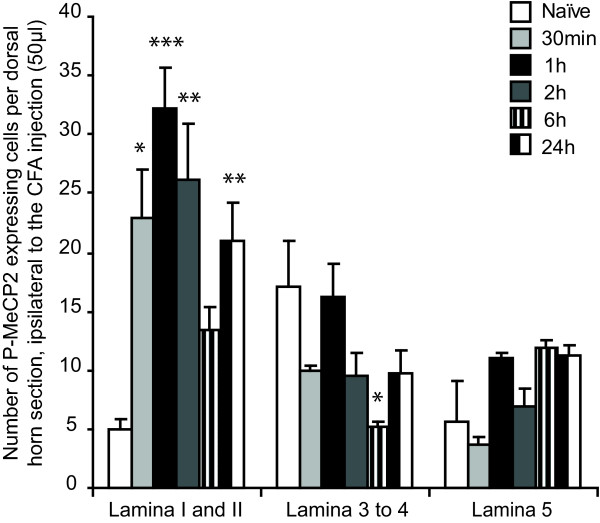
**Time course of P-MeCP2 expression in the superficial dorsal horn after CFA injection**. CFA injection in the hind paw significantly increased MeCP2 phosphorylation in the superficial dorsal horn, ipsilateral to the injection. Changes in P-MeCP2 expression were seen exclusively in Lamina I and II, with the exception of expression in Lamina 3 to 4 at 6 h. Data show mean ± standard error of the mean. *P < 0.05, **P < 0.001, ***P < 0.0001, always *vs *naïve within the same dorsal horn area.

**Figure 2:**
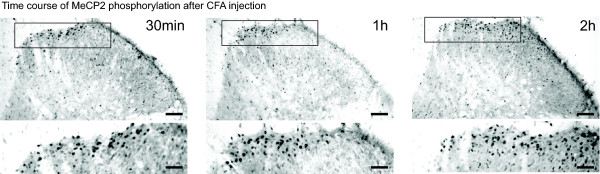
**Expression of P-MeCP2 in the superficial dorsal horn after CFA injection**. Pictures show typical P-MeCP2 immunoreactivity in the dorsal horn, ipsilateral to the injection, 30 min, 1 h and 2 h after CFA injection in the hind paw. Scale bars, top: 100 μm; bottom: 50 μm.

We followed the changes in P-MeCP2 for up to 24 h after injection of CFA in the hind paw (N = 3 at each time point). Changes were only seen ipsilateral of the injection and were mainly restricted to laminae I-II (see below and Figure 1 and Figure 2). The only other significant changes across the dorsal horn were seen in lamina 3–4, 6 h after CFA injection (significant decrease in P-MeCP2 compared to naïve from 17 ± 4 to 5.2 ± 0.4 immunopositive nuclei).

**Figure 3:**
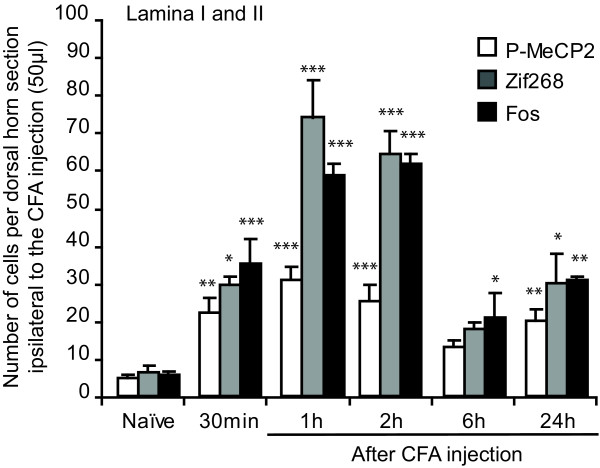
**Expression of P-MeCP2, Zif268 and Fos in the superficial dorsal horn after CFA injection**. There was a significant effect of CFA on MeCP2 phosphorylation and Zif268 and Fos expression, with maximum changes at 1 h and 2 h. Data show mean ± standard error of the mean. *P < 0.05, **P < 0.001, ***P < 0.0001, always *vs *naïve for each of the transcription regulator.

**Figure 4:**
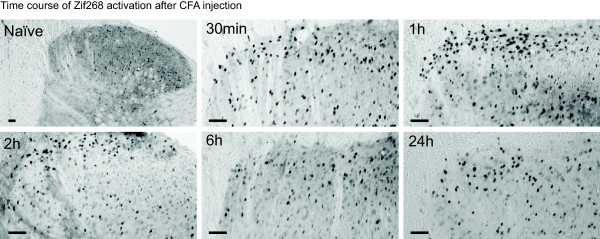
**Time course of Zif268 expression in the superficial dorsal horn after CFA injection**. Pictures show typical Zif268 immunoreactivity in the dorsal horn, ipsilateral to the injection, before (naïve) and 30 min, 1 h, 2 h, 6 h and 24 h after CFA injection in the hind paw. Scale bars, 50 μm.

We compared the expression of P-MeCP2 with that of Zif268 and Fos in laminae I-II across all time points (Figure 3 and Figure 4; N = 3/5 at each time point). The three transcriptional regulators showed a similar pattern of activation in the medial area of the superficial dorsal horn. There was a significant increase in P-MeCP2, Zif268 and Fos from 30 min post CFA. Strikingly, for all genes, the maximum changes were seen within 1 h to 2 h after CFA administration. In laminae I-II, P-MeCP2 increased from 5 ± 1 to 32 ± 3 immunopositive nuclei (*P *< 0.001), Zif268 from 7 ± 1 to 74 ± 10 (*P *< 0.001) and Fos from 6 ± 1 to 61 ± 3 (*P *< 0.001) (Figure 3). There was no significant difference in the levels of P-MeCP2 between 1 h and 2 h, nor of Zif268 or Fos. Although the levels of P-MeCP2 and Zif268 seen 6 h after CFA were no different from that in naïve animals, there was a significant upregulation in the levels of P-MeCP2, Zif268 and Fos 24 h after CFA (increase to 21 ± 3 immunopositive nuclei (*P *< 0.01), 31 ± 8 immunopositive nuclei (*P *< 0.05) and 32 ± 1 (*P *< 0.01) respectively).

### P-MeCP2 is co-expressed in neurones with Zif268 and Fos

**Figure 5:**
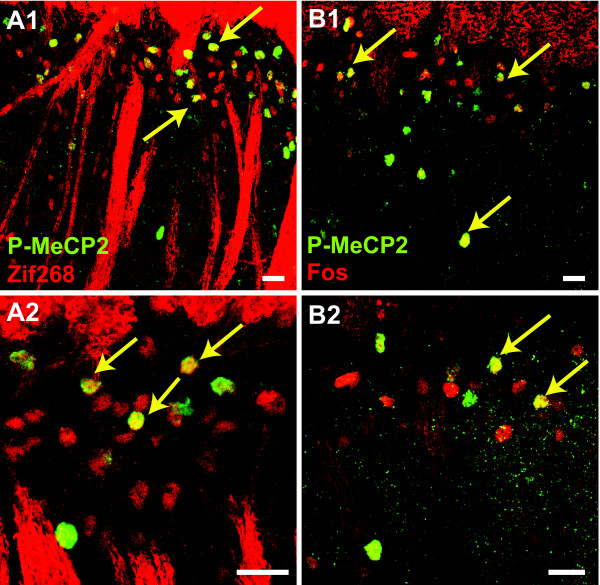
**P-MeCP2 is expressed in sub-populations of Zif268 and Fos expressing neurones after CFA injection**. Tissue was taken 1 h after CFA injection. Spinal cord sections were double-labelled with antibodies against P-MeCP2 (green) and ***A***/ Zif268 (red) or ***B***/ Fos (red). Double labelling resulted in yellow staining (arrows). Images were taken using confocal microscopy. Scale bars, 20 μm.

We next investigated the potential overlap in neuronal population expressing these transcription regulators in laminae I-II. Tissue was taken at 1 h and 2 h after CFA and sections were double-labelled for P-MeCP2 and Zif268, and P-MeCP2 and Fos (N = 2/4 in each group). All images of double stained dorsal horn tissue were acquired by confocal microscopy using a laser scanning microscope (Leica TCS NT SP). A large population of neurones expressed both P-MeCP2 and Zif268, and P-MeCP2 and Fos (Figure 5A and 5B). Interestingly, there was no difference in the ratio of P-MeCP2 neurones expressing Zif268 at 1 h and 2 h (78% ± 5% and 76% ± 5%, respectively) whereas the number of P-MeCP2 neurones expressing Fos increased slightly with time (82% ± 2% at 1 h and 90% ± 1% at 2 h, *P *< 0.05).

### Descending serotonergic inputs are essential for the full expression of P-MeCP2, Zif268 and Fos in the dorsal horn

**Figure 6:**
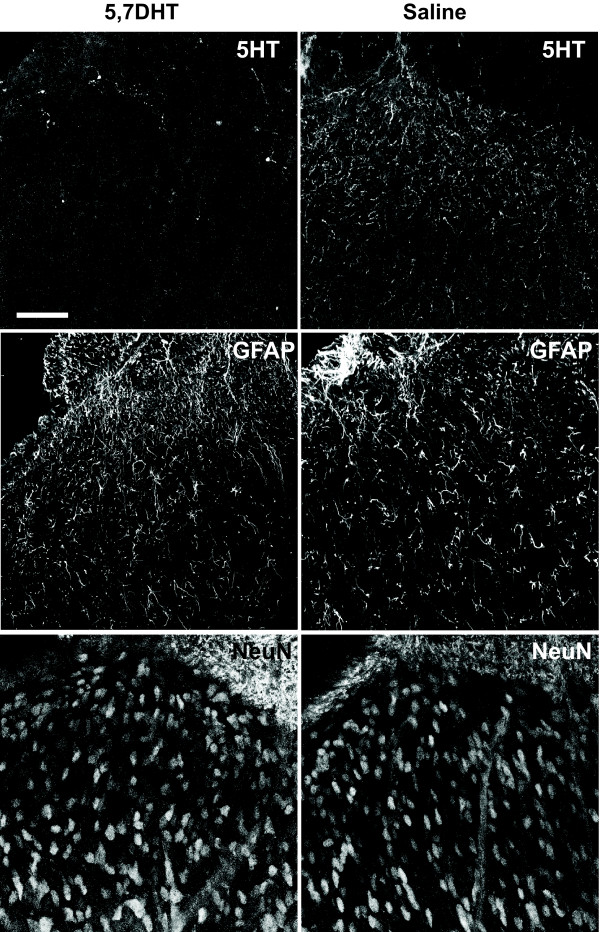
**Intrathecal 5,7-DHT causes serotonin depletion but no glial proliferation or cell death**. Tissue was taken 7 days after 5,7-DHT treatment and dorsal horn sections were stained for 5-HT, GFAP and NeuN. 5-HT staining confirmed a complete depletion of 5-HT in the superficial dorsal horn 7 days after 5,7-DHT, and GFAP and NeuN staining indicated that, at the concentration used in this study, 5,7-DHT did not cause glial proliferation or hypertrophy or abnormal cell death. Scale bar, 50 μm.

**Figure 7:**
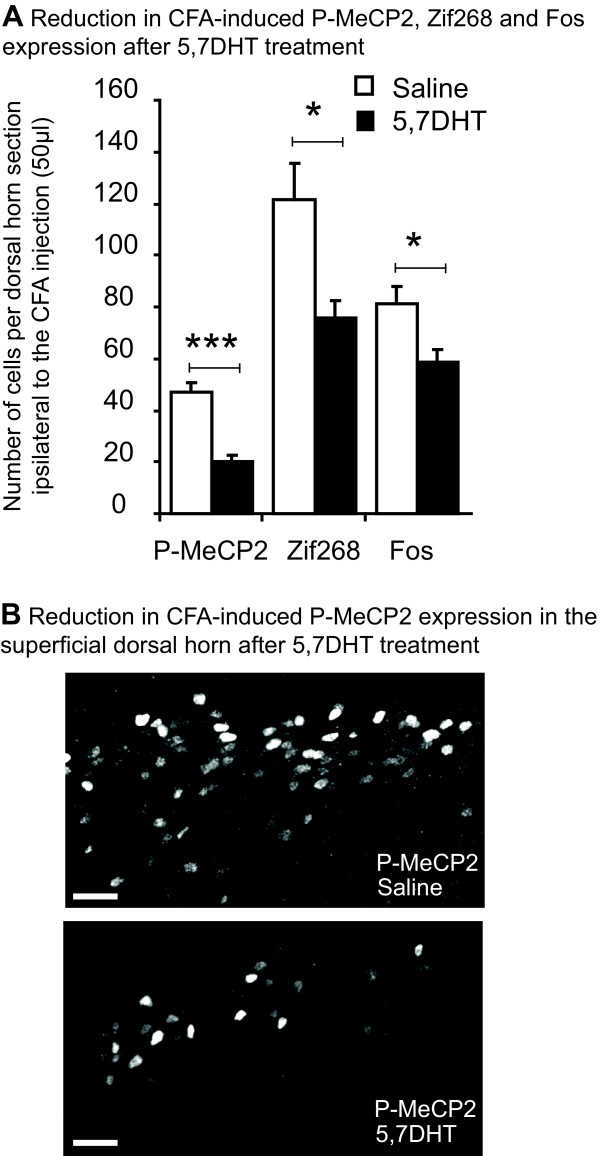
**5,7-DHT treatment reduces CFA-induced P-MeCP2, Zif268 and Fos expression**. Seven days after 5,7-DHT or saline treatment, animals received CFA and were sacrificed after 1 h. ***A***/ P-MeCP2, Zif268 and Fos expression 1 h after CFA injection in the hind paw were significantly reduced by 5,7-DHT. N = 4/6 in each group. Data show mean ± standard error of the mean; *P < 0.05, ***P < 0.0001. ***B***/ Pictures show typical P-MeCP2 immunoreactivity in the dorsal horn, ipsilateral to the injection, 1 h after CFA injection in the hind paw. 5,7-DHT reduced P-MeCP2 immunoreactivity compared to saline, especially in the medial area of the superficial dorsal horn. Scale bars, 40 μm.

Animals were separated in to 2 groups, one receiving an intrathecal injection of saline and the other of 5,7-DHT (N = 4/6 in each group). Seven days later, animals received CFA and were sacrificed after 1 h. 5-HT staining showed complete depletion of 5-HT in the superficial dorsal horn after 5,7-DHT, and GFAP and NeuN staining indicated that 5,7-DHT did not cause glial proliferation or hypertrophy or abnormal cell death (Figure 6). When animals received 5,7-DHT, there was a significant reduction compared with saline in expression of P-MeCP2 ipsilateral to the CFA injection (47 ± 4 *vs *20 ± 2 immunopositive nuclei; F_1,10 _= 37, *P *< 0.001), Zif268 (121 ± 14 *vs *76 ± 7; F_1,10 _= 11, *P *< 0.05) and Fos (81 ± 7 *vs *59 ± 5; F_1,10 _= 8, *P *< 0.05) in laminae I-II (Figure 7A and 7B).

**Figure 8:**
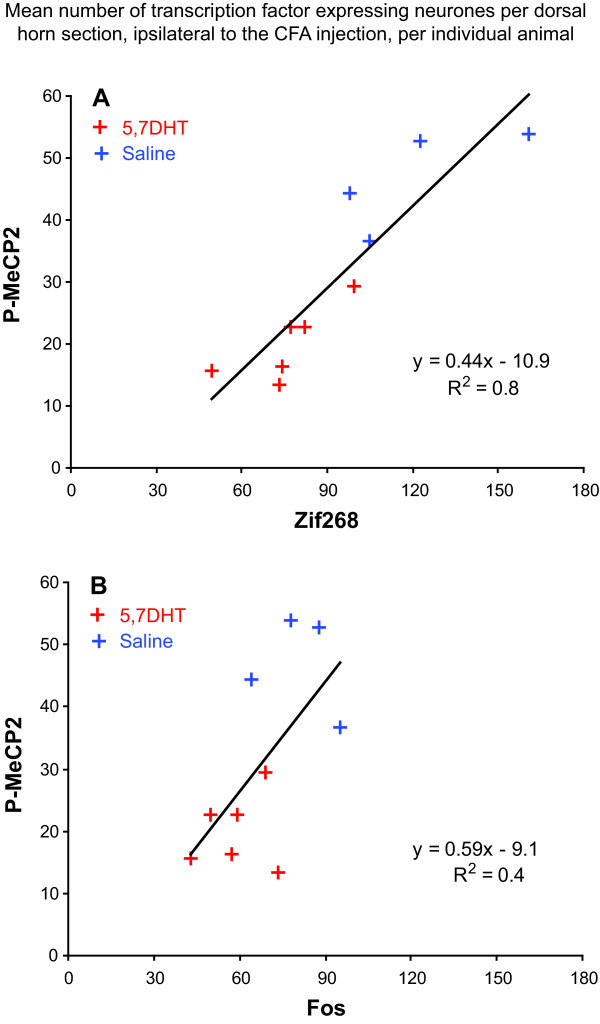
**The correlation between P-MeCP2 and Zif268 regulation is stronger than that between P-MeCP2 and Fos**. The graphs represent the mean number of Zif268 (***A***) or Fos (***B***) expressing neurones per dorsal horn section, ipsilateral to the CFA injection, per individual animal, against the number of P-MeCP2 immuno-positive neurones. The correlation is considerably stronger between P-MeCP2 and Zif268 (correlation coefficient: R^2 ^= 0.8) than that between P-MeCP2 and Fos (R^2 ^= 0.4), suggesting a parallel regulation for P-MeCP2 and Zif268.

When we plotted the mean number per dorsal horn section, ipsilateral to the CFA injection, of P-MeCP2 positive nuclei against the number of Zif268 or Fos positive nuclei for individual animals for this experiment (N = 10), the correlation was stronger between P-MeCP2 and Zif268 (correlation coefficient: R^2 ^= 0.8) than between P-MeCP2 and Fos (R^2 ^= 0.4), suggesting a parallel regulation for P-MeCP2 and Zif268 (Figure 8).

### Depletion of serotonergic axons in the dorsal horn reduces mechanical sensitivity but does not alter the thermal hyperalgesia that develops after CFA

**Figure 9:**
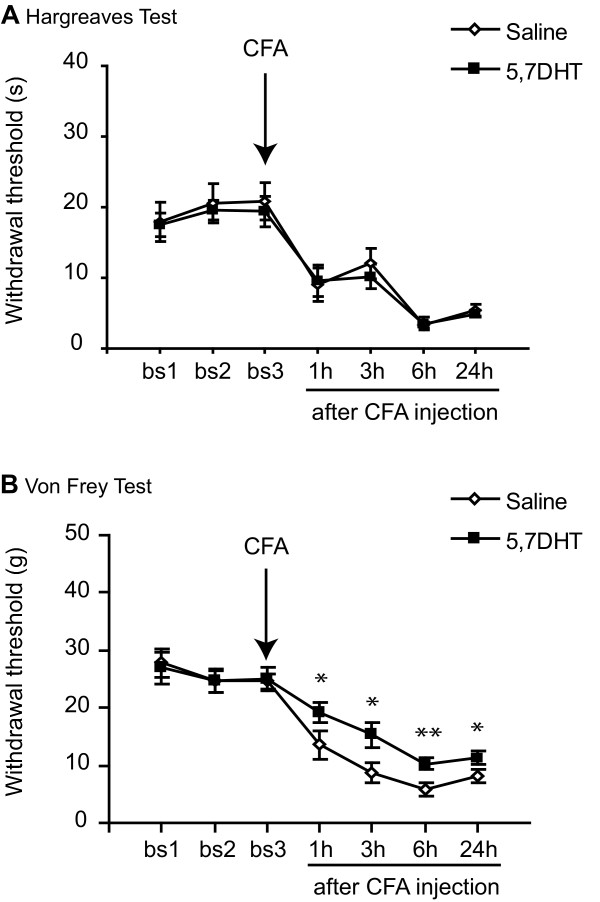
**5,7-DHT attenuates mechanical sensitivity but not thermal sensitivity**. Seven days after 5,7-DHT or saline treatment, animals received CFA in the hind paw. ***A***/ 5,7-DHT did not change the increased thermal sensitivity seen after CFA injection in the hind paw as measured by the Hargreaves test. N = 5/6.***B***/ 5,7-DHT significantly attenuated the inflammation-induced decrease in mechanical threshold measured by the automatic Von Frey from 1 h after CFA injection. The response was still attenuated 24 h later. N = 7/8. For ***A***, and ***B***, data show mean ± standard error of the mean; *P < 0.05, **P < 0.001.

We tested the hypothesis that the increase in pain sensitivity seen very early after the induction of an inflammation state could be reduced by ablation of descending serotonergic pathways. For this, animals were separated in 2 groups, one receiving an intrathecal injection of saline and the other of 5,7-DHT. These 2 groups were further divided into 2 groups, one dedicated to thermal testing (N = 5/6) and the other to mechanical testing (N = 7/8) (animals were never tested in 2 different modalities). Animals were all tested before and after intrathecal injections and mechanical and heat thresholds were similar in the 2 treatment groups before CFA injection. Seven days after the intrathecal treatment, animals received CFA into the hind paw and their thresholds were measured 1 h, 3 h, 6 h and 24 h after injection. There was no effect of 5,7-DHT on the heat threshold (Figure 9A). However depletion of 5-HT significantly attenuated the decrease in mechanical threshold seen after the induction of inflammation from 1 h after CFA. The response was still attenuated 24 h later (overall ANOVA: F_1,13 _= 7.2, *P *< 0.05) (Figure 9B).

## Discussion

Noxious stimulation of the periphery leads to a large number of rapid post-translational and transcriptional changes in dorsal horn neurones. We show that the activation or expression of the transcription factors MeCP2, Zif268 and Fos after primary afferents stimulation depend on convergent input from primary afferents and descending pathways from the brain.

### Time course of molecular and behavioural changes in a model of inflammation

The phosphorylation of MeCP2 in dorsal horn neurones following CFA was surprisingly slow compared to other transcription regulators such as cAMP response element-binding (CREB) and extracellular signal-regulated kinase (ERK) which are phosphorylated in dorsal horn neurones within 5 min of peripheral nociceptive stimulation [15,16]. However, the time course of phosphorylation of MeCP2 closely paralleled that of Zif268 and Fos expression, suggesting a common time course of regulation of gene expression for these 3 transcription factors. Moreover, there was a strong correlation between the changes in transcription factors activation and animal behaviour from 30 min to 2 h after CFA, supporting the idea that macromolecular changes could contribute to this early behavioural change.

Early post-translational changes are clearly important in the initial phases of sensitization [17,18]. However, translation following new gene transcription or from mRNA already present in neuronal cell bodies and their processes is necessary for maintaining the central sensitization that follows peripheral noxious stimulation. Such process can also participate to the early phases of sensitizition. Indeed, recent studies showed that protein synthesis in the dorsal horn contributes to the increase in mechanical sensitivity seen after noxious stimulation from *30 min *post stimulation [19,20].

It seems likely that the expression of P-MeCP2, Zif268 and Fos is part of an early cascade of molecular events that lead to changes in gene expression required for both the initiation and maintenance of pain states. This is supported by our recent study where the mechanical hypersensitivity seen in an animal model of arthritis remained maximum from 6 h to 7 days after CFA injection, while the pattern of gene expression differed throughout the time course studied [7].

### Molecular changes in superficial dorsal horn modulated by descending pathways

We found that phosphorylation of MeCP2 that follows CFA injection was decreased by serotonin depletion. We have previously reported that, after induction of peripheral joint inflammation, MeCP2 was phosphorylated in rat superficial dorsal horn and lamina I/III projection neurones, and that expression of SGK1, a protein under transcriptional repression by MeCP2, also expressed in lamina I/III projection neurones, increased in the superficial dorsal horn in an inflammatory pain state [7]. Antisense knock down of this protein delayed the onset of the increased mechanical sensitivity seen in a state of inflammation, strongly supporting a role for MeCP2 in the initiation, and possibly maintenance, of pain states. In the present study, we were able to show that serotonin depletion attenuated both MeCP2 activation and the increased mechanical sensitivity that follows CFA injection, again strengthening the correlation between transcription factors activation and changes in behaviour.

We also report that serotonin depletion reduced inflammation-induced Fos. Although controversial, recent research using antisense approaches to deplete dorsal horn Fos protein levels has largely indicated that Fos induction leads to increased pain sensitivity particularly during the second phase of the formalin test [21-23]. Moreover, up-regulation of dynorphin A, which has been shown to be pronociceptive by acting at the bradykinin receptors (B1/2) on primary afferents [24], was linked to Fos expression [25].

Expression of inflammation-induced Zif268 was also attenuated after serotonin depletion, in parallel with mechanical sensitivity. Nociceptive stimulus-specific expression of Zif268 was shown to be required for the maintenance of long-term potentiation (LTP) and spatial learning in the hippocampus [8]. We also recently proposed a role for Zif268 in maintaining the increased sensitivity of spinal neurones in inflammatory pain condition [9].

Here, we found that a large population of P-MeCP2 immunopositive nuclei were also expressing Fos (82–90%) and Zif268 (76–78%). The possibility that Fos is a target gene for MeCP2 has been excluded by previous *in vitro *studies [6] and recent studies of gene expression changes in brain tissue of MeCP2 deficient mice failed to report any changes in Fos and Zif268 mRNA [26]. However, it is well accepted that MeCP2 activity is extremely cell type specific and therefore we cannot exclude a possible direct interaction between MeCP2 and Fos or/and Zif268 promoter region within superficial dorsal horn neurones. Evidence also suggests that an increase in P-MeCP2 may lead to a persistent up-regulation of Fos as seen here at 6 h and 24 h following CFA. Phosphorylation of MeCP2 has been shown to de-repress BDNF expression [27], an increase in BDNF mRNA has been found in the dorsal horn after CFA injection [28] and intrathecal BDNF challenge has been shown to induce the transcription of *c-fos *within 90 min [29].

### Lamina I projecting neurones

Crucially, P-MeCP2, Zif268 and Fos are all up-regulated in lamina I, NK1 positive, projection neurones following peripheral noxious stimulation: after peripheral inflammation 47% and 27% of projection neurones expressed Zif268 [30] and P-MeCP2 [7] respectively. However only 10% of NK1 expressing neurones expressed Fos, irrespectively of the noxious stimulation (skin, joint, muscle, inflammation and nerve injury) [31], strongly suggesting that Zif268 and P-MeCP2 are more generally expressed in NK1-positive projections neurones than Fos.

Lamina I neurones project to the brainstem and thalamus, indirectly activate descending pathways to the dorsal horn and are essential for the full development of pain states [32,33]. Our present findings suggest that the transcription regulators that contribute to the plasticity of projection neurones that engage descending pathways are themselves regulated by these descending controls. This suggests that the initiation and/or maintenance of pain states depend on feedback loop mechanisms and support the idea that regulation of gene expression in lamina I projection neurones is key to the molecular control of pain sensitivity.

### Descending serotonergic control of pain thresholds

We report that ablation of serotonergic innervation of the lumbar dorsal horn attenuates increased mechanical but not thermal sensitivity in an inflammatory pain state. How might this dissociation have been achieved? Inflammatory pain states are dependent upon changes in both peripheral nociceptors and neurones in the superficial dorsal horn. In other words, the amplification of the response to thermal or mechanical stimulation of inflamed tissue can be due to peripheral or/and central sensitization. Previous research has identified clear peripheral thermal sensitization of primary afferents nociceptors following inflammation but mechanical sensitization of cutaneous afferents has been rather more controversial. Andrew and Greenspan (1999) conclude that mechanical sensitization of primary afferents by inflammatory agents does indeed occur but is only uncovered by supramaximal mechanical stimulation [34]. In the present study, behavioural assessment did not use supramaximal stimulation and most of the amplification of the response to mechanical stimulation of inflamed tissue would have been largely due to central sensitization. Our results therefore suggest that central sensitization that leads to increased mechanical sensitivity is depressed by serotonergic lesions. Whether thermal sensitivity is modulated by central sensitization in the same way would be more difficult to assess with behavioural techniques but distant secondary thermal changes are reported to be sensitive to manipulations of the RVM [35-37].

### Serotonin Receptor Mechanisms

There is evidence for both inhibitory *and *facilitatory serotonergic and non-serotonergic inputs to the dorsal horn [2,10,38,39]. Our present findings, along with a number of previous reports [1,12,40,41], highlight the importance of a serotonergic descending facilitatory system. Facilitatory actions of spinal serotonin have been shown to engage the 5-HT3 receptor (5-HT3-R) [12,40] which is predominantly found on primary afferent axons and axons of dorsal horn neurones [42]. However, the mechanical hypersensitivity that develops after CFA injection did not differ between 5-HT3-R mutant and wild-type mice [43]. Other serotonergic receptors have also been linked with descending serotonergic facilitation and would also deserve attention: *e.g*. 5-HT2A [44], 5-HT7 [45], 5-HT1A, 5-HT2C and 5-HT4 receptors [13].

The balance between descending inhibition and excitation is a crucial one: descending facilitation must be tonic over long periods of time to maintain the sensitivity around the site of injury during healing, preventing further damage, while descending inhibition is crucial for the short lasting expression of endogenous analgesia required during conflict or when an animal in pain needs to move to acquire food and water.

## Competing interests

The authors declare that they have no competing interests.

## Authors' contributions

SMG and SPH conceived, designed and performed the experiments, analysed the data and wrote the manuscript. VF designed and performed part of the experiments. KKT carried out some of the immunohistochemistry. All authors read and approved the final manuscript.
